# Elevated Adiponectin Antibody Levels in Sera of Patients with Atherosclerosis-Related Coronary Artery Disease, Cerebral Infarction and Diabetes Mellitus

**DOI:** 10.5772/63218

**Published:** 2016-04-07

**Authors:** Takaki Hiwasa, Xiao-Meng Zhang, Risa Kimura, Mikiko Ohno, Po-Min Chen, Eiichiro Nishi, Koh Ono, Takeshi Kimura, Ikuo Kamitsukasa, Takeshi Wada, Akiyo Aotsuka, Seiichiro Mine, Hirotaka Takizawa, Koichi Kashiwado, Minoru Takemoto, Kazuki Kobayashi, Harukiyo Kawamura, Ryoichi Ishibashi, Koutaro Yokote, Rika Nakamura, Go Tomiyoshi, Natsuko Shinmen, Hideyuki Kuroda

**Affiliations:** 1 Department of Biochemistry and Genetics, Graduate School of Medicine, Chiba University, Chiba, Japan; 2 Department of Cardiovascular Medicine, Graduate School of Medicine, Kyoto University, Kyoto, Japan; 3 Department of Neurology, Chiba Rosai Hospital, Chiba, Japan; 4 Department of Internal Medicine, Chiba Aoba Municipal Hospital, Chiba, Japan; 5 Department of Neurological Surgery, Chiba Prefectural Sawara Hospital, Chiba, Japan; 6 Department of Neurological Surgery, Graduate School of Medicine, Chiba University, Chiba, Japan; 7 Port Square Kashiwado Clinic, Kashiwado Memorial Foundation, Chiba, Japan; 8 Department of Neurology, Kashiwado Hospital, Chiba, Japan; 9 Department of Clinical Cell Biology and Medicine, Graduate School of Medicine, Chiba University, Chiba, Japan; 10 Medical Project Division, Research Development Center, Fujikura Kasei Co., Saitama, Japan

**Keywords:** Atherosclerosis, Cardiovascular Disease, Cerebral Infarction, Diabetes Mellitus, Antibody Biomarker

## Abstract

Adiponectin secreted from the adipocytes plays pleiotropic, anti-atherosclerotic roles, such as enhancement of insulin secretion and an increase in energy expenditure. The measurement of levels of circulating adiponectin is useful to evaluate the progression of atherosclerosis-related diseases, such as coronary artery disease (CAD), cerebral infarction (CI) and diabetes mellitus (DM). We examined the serum antibody levels against recombinant adiponectin protein via the amplified luminescent proximity homogeneous assay-linked immunosorbent assay (AlphaLISA) method. The results revealed that the antibody levels were significantly higher in patients with CAD, CI and type 2 DM, than in healthy donors. Receiver operating curve analysis showed that the sensitivity was in a range of 41–48% for CAD, CI and DM. Thus, the serum anti-adiponectin antibody levels could be a common marker for atherosclerosis-related diseases.

## 1. Introduction

Adiponectin is a peptide hormone that plays a variety of roles in glucose and lipid metabolism, diabetes (DM) and metabolic syndrome [1–4]. The circulating adiponectin level is negatively correlated with obesity, coronary artery disease (CAD) and metabolic disorders [5,6]. Adiponectin reduces atherosclerosis in apolipoprotein E-deficient mice [7]. Furthermore, exacerbation of heart failure was observed in adiponectin-deficient mice [8]. AdipoR1 and AdipoR2 are the major typical receptors of adiponectin, and depletion of both receptors leads to insulin resistance and glucose intolerance [9]. These results support the idea that adiponectin has a causal role in suppressing the development of diabetes mellitus (DM), CAD and atherosclerosis.

We have recently reported novel atherosclerosis-related antibody markers, such as antibodies against RPA2 for stroke [10], antibodies against SOSTDC1 and TUBB2C for CI and DM [11,12], and antibodies against ATP2B4 and BMP-1 for atherosclerosis-related diseases, such as CI, CAD, DM and chronic kidney disease [13]. Abnormality of the blood vessels may result in the leaking out of the antigenic proteins that can produce these antibodies. In the present study, the levels of autoantibody against those of adiponectin were examined. Our results showed that the antibody levels were associated with CAD, CI and DM.

## 2. Methods

### 2.1 Sera of patients and healthy donor (HD) subjects

The Local Ethical Review Board of Chiba University, Graduate School of Medicine (Chiba, Japan) as well as those of co-operating hospitals approved the study. Sera were collected from patients after they had provided written informed consent. Each serum sample was centrifuged at 3,000 × g for 10 min, and supernatants were stored at −80°C until use. The serum samples of CAD, including acute myocardial infarction (AMI), were obtained from the Kyoto University Hospital. The samples of acute cerebral infarction (ACI) were obtained from Chiba Rosai Hospital and Chiba Aoba Municipal Hospital. The samples of type 2 DM were obtained from Chiba University Hospital. Sera from HD subjects were obtained from Chiba University, Chiba Prefectural Sawara Hospital and Port Square Kashiwado Clinic.

### 2.2 Amplified Luminescence Proximity Homogeneous Assay (AlphaLISA)

The antigen used for the analysis of CAD and ACI specimens was recombinant His-tag-conjugated, full-length adiponectin protein (ProSpec-Tany TechnoGene Ltd., Ness Ziona, Israel). The antigen used for the analysis of age-matched AMI and DM specimens was His-tag-conjugated adiponectin peptide (amino acids 108–244) (ATGen Co. Ltd., Seongnam, South Korea). AlphaLISA was performed using 384-well microtitre plates (white opaque OptiPlate™, Perkin Elmer, Waltham, MA) containing 2.5 μL of 1/100-diluted sera and 2.5 μL of His-tag adiponectin (10 μg/mL) in AlphaLISA buffer (25 mM HEPES, pH 7.4, 0.1% casein, 0.5% Triton X-100, 1 mg/mL dextran-500 and 0.05% Proclin-300). The reaction mixture was incubated at room temperature for 6–10 h. Nickel-chelate donor beads (2.5 μL of 40 μg/mL) and anti-human IgG-conjugated acceptor beads (2.5 μL of 40 μg/mL) were then added and incubated further for seven to 21 days at room temperature in the dark. The chemical emission was read on an EnSpire Alpha microplate reader (PerkinElmer) as previously described [11–13]. Specific reactions were calculated by subtracting Alpha values without antigens from the values of adiponectin proteins.

### 2.3 Statistical Analyses

Student's *t* test and the Mann-Whitney U test were used to determine the significance of the differences between the two groups. The correlation was examined via Spearman's correlation analysis. All statistical analyses were carried out using GraphPad Prism 5 (GraphPad Software, La Jolla, CA). The predictive values of markers for diseases were assessed by receiver operating curve (ROC) analysis, and the cut-off values were set at the values that maximize the sums of the sensitivity and specificity. All tests were two-tailed and a *P* value below 0.05 was considered significant.

## 3. Results

### 3.1 Levels of adiponectin antibodies (adiponectin-Abs) are associated with CAD

**Figure 1.**
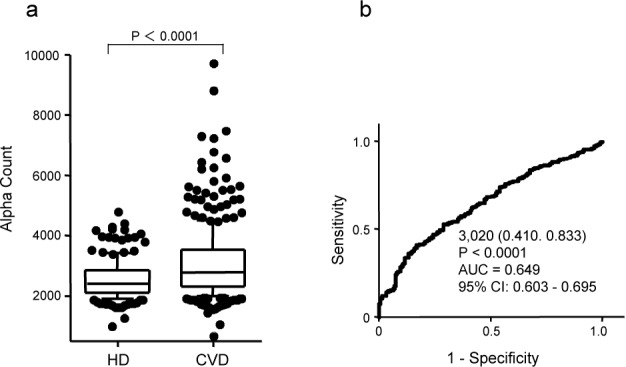
Comparison of serum adiponectin antibodies (adiponectin-Ab) levels between the healthy donor (HD) subjects and patients with coronary artery disease (CAD). Serum antibody levels examined by AlphalLISA are shown by a box-whisker plot (a). The box plots display the 10th, 20th, 50th, 80th and 90th percentiles. P values as compared to the HD specimens are shown. Receiver operating curve (ROC) analysis was carried out for assessing the ability of adiponectin-Abs to detect CAD (b). Numbers in the curves indicate cut-off values of marker levels and those in parentheses indicate sensitivity (left) and specificity (right). P values of ROC analysis, areas under the curve (AUC) and a 95% confidence interval (CI) are also shown.

We examined the relationship between adiponectin-Abs and CAD, which included sera from AMI and unstable angina patients obtained from Kyoto University Hospital. HD subjects from Kashiwado Clinic and Chiba Prefectural Sawara Hospital were selected as those who had no apparent abnormality on regular health check-ups, including those based on MRI examination. The average ages of the HD subjects and patients were 49.8 and 66.6, respectively. The results of AlphaLISA showed that the levels of serum adiponectin-Abs were significantly higher in patients with CAD than those in the HD subjects (Figure 1a). When the cut-off value was determined as the average + 2SD of the HD specimens, the positive rates of adiponectin-Abs in HD subjects and patients with CAD were 6.8% and 17.7%, respectively (Table 1). Receiver operating curve (ROC) analysis was carried out to evaluate the ability of these markers to detect CAD. The areas under the curve (AUCs) of adiponectin-Abs for CAD were 0.649 (95% CI: 0.603–0.695) (Figure 1b). When the cut-off value of the adiponectin-Ab level was determined to be 3,020, the sensitivity and specificity of the antibody level for the diagnosis of CAD were calculated to be 41.0% and 83.3%, respectively.

**Table 1.** Comparison of serum anti-adiponectin antibody levels between the HD subjects and ACI, age-matched ACI, CAD, age-matched AMI and age-matched type 2 DM patients, examined by AlphaLISA. The table shows averages, SDs, cut-off values (average + 2SD), total sample numbers, the numbers of positive sera of which antibody levels were higher than the cut-off value and the positive rates (%) of the HD subjects; averages, SDs, total sample numbers, numbers of positive sera of which the antibody levels were higher than the cut-off value and the positive rates (%) of patients; and P values of the statistical comparison between the HD subjects and patients.

		CAD	AMI, age-matched	ACI	ACI, age-matched	DM, age-matched
HD	Average	2,526	1,310	2,535	2,492	959
	SD	640	455	368	383	673
	Cut-off Value	3,805	2,219	3,272	3,257	2,306
	Total No.	191	127	72	44	128
	Positive No.	13	6	2	2	6
	Positive Rate (%)	6.8%	4.7%	2.8%	4.5%	4.7%

Patient	Average	3,087	1,679	3,045	2,813	1,392
	SD	1,368	550	1,078	896	1,076
	Total No.	378	128	286	54	128
	Positive No.	67	18	87	11	12
	Positive Rate (%)	17.7%	14.1%	30.4%	20.4%	9.4%

P value	(Patient vs. HD)	6.2E-11	1.6E-08	1.5E-10	2.4E-02	1.5E-04

We then compared the antibody levels between the HD subjects and age-matched patients with AMI. The average ages of the HD subjects and the patients were 57.9 and 57.2, respectively. The levels of adiponectin-Abs were also significantly higher in patients with AMI as compared with age-matched HD subjects (Table 1). The positive rates in the HD subjects and patients with AMI were 4.7% and 14.1%, respectively. ROC analysis revealed that the AUC of adiponectin-Abs in patients with AMI was 0.641 (95% CI: 0.574–0.709). The sensitivity and specificity of the antibody level for diagnosis of AMI were calculated to be 47.7% and 74.2%, respectively.

### 3.2 Levels of adiponectin-Abs are increased in patients with ACI

**Figure 2.**
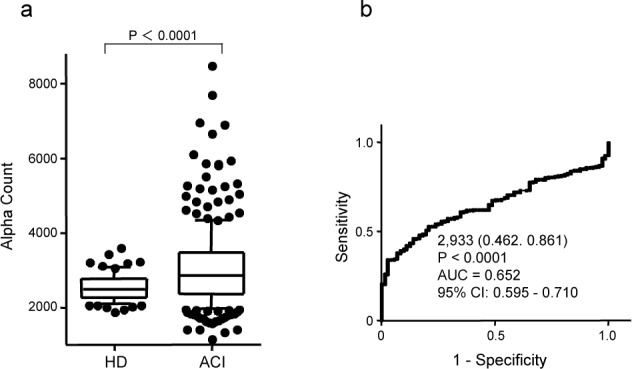
Comparison of serum adiponectin-Ab levels between the HD subjects and patients with acute cerebral infarction (ACI). Serum antibody levels examined by AlphalLISA are shown by a box-whisker plot as described in the legend to Figure 1. The results were also evaluated by ROC analysis (b).

We next examined adiponectin-Ab levels in the sera of the HD subjects obtained from Chiba University and Chiba Prefectural Sawara Hospital, and in the sera of patients with ACI obtained from Chiba Rosai Hospital and Chiba Aoba Municipal Hospital. The average ages of the HD subjects and the patients were 44.3 and 69.3, respectively. (Figure 2a). The positive rates of adiponectin-Abs in the HD subjects and patients with ACI were 2.8% and 30.4%, respectively (Table 1). ROC analysis revealed that the AUC of adiponectin-Abs for ACI was 0.652 (95% CI: 0.595–0.710; Figure 2b). When the cut-off value of the adiponectin-Ab level was determined to be 2,933, the sensitivity and specificity of the antibody level for the diagnosis of ACI were 46.2% and 86.1%, respectively.

Because the average ages of the HD subjects and the patients were different, the antibody levels of age-matched specimens were compared after depletion of the appropriate samples. The average ages of the HD subjects and the patients selected were 50.8 and 51.0, respectively. Despite the decrease in sample numbers examined, the levels of adiponectin-Abs were still significantly higher in patients with ACI than in the HD subjects (P = 0.024; Table 1). The positive rates of adiponectin-Abs in the HD subjects and patients with ACI were 4.5% and 20.4%, respectively.

### 3.3 Levels of adiponectin-Abs are related to DM

**Figure 3.**
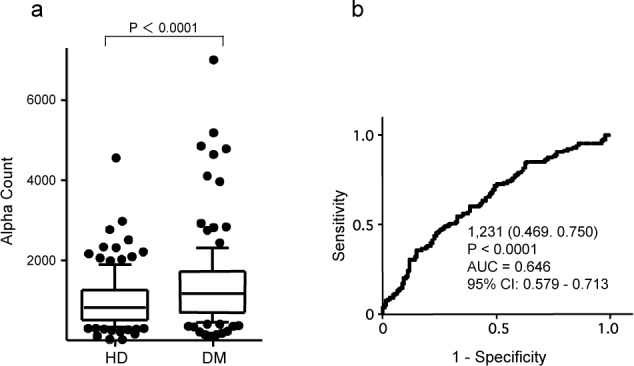
Comparison of serum adiponectin-Ab levels between the HD subjects and patients with type 2 diabetes mellitus (DM). Serum adiponectin-Ab levels examined by AlphalLISA are shown by a box-whisker plot as described in the legends of Figure 1 (a). The results were also evaluated by ROC analysis (b).

Because atherosclerosis is closely related to type 2 DM, we then compared the specimens of the HD subjects and age-matched patients with type 2 DM obtained from Kashiwado Clinic and Chiba University Hospital, respectively. The average ages of the HD subjects and the patients were 57.8 and 58.5, respectively. The levels of adiponectin-Abs were higher in patients with DM than in the HD subjects (Figure 3a). The positive rates of adiponectin-Abs in the HD subjects and patients with DM were 4.7% and 9.4%, respectively (Table 1). ROC analysis revealed that the AUC of adiponectin-Abs for DM was 0.646 (95% CI: 0.579–0.713) (Figure 3b). The sensitivity and specificity of the antibody levels for the diagnosis of DM were calculated to be 46.9% and 75.0%, respectively.

## 4. Discussion

Recent studies have shown that autoantibodies develop and increase, not only in instances of autoimmune diseases and cancer but also in other metabolic and vascular diseases, such as autoantibodies to oxidized low-density lipoprotein and β2-glycoprotein I in atherosclerosis [14,15], heat shock proteins (Hsps) in acute cardiovascular diseases [16], Hsp60 in stroke [17] and GAD in DM [18,19]. We have reported that RPA2 antibodies increase in stroke [10]. SOSTDC1 and TUBB2C antibodies are associated with CI and DM [11,12]. ATP2B4 and BMP-1 antibodies increase in atherosclerosis-related diseases, such as CI, CAD, DM and chronic kidney disease [13]. Adiponectin is a protein that is closely and inversely associated with glucose tolerance and atherosclerosis [1–9]. Thus, we examined the presence of autoantibodies against adiponectin by the highly sensitive AlphaLISA method, which produces highly reproducible and stable results because it makes plate washing unnecessary. This is the first report that confirms the presence of adiponectin-Abs in sera. The levels of adiponectin-Abs were significantly higher in patients with ACI, AMI and DM than in the HD subjects (Figures 1–2, Table 1).

When the cut-off values were determined as the average + 2SD of the HD specimens, the positive rates of ACI were somewhat higher than those of CAD (Table 1). However, when the age-matched specimens of HD and patients were compared, the positive rates were not apparently different. Furthermore, AUC values and percentages of sensitivity and specificity calculated by ROC analysis were quite similar among CAD, ACI and DM (Figures 1–3). Taken together, this suggests that adiponectin-Abs are almost equally associated with these atherosclerosis-related diseases.

Spearman's correlation analysis was performed between the adiponectin-Ab levels and the patients' data, including age, gender, height, weight, BMI and blood pressure, using the sera of patients with ACI from Rosai Hospital. Unexpectedly, the antibody levels failed to show a significant correlation with the patients' data (data not shown). Therefore, the adiponectin-Ab levels may simply reflect whether or not the patients suffer from atherosclerosis-related diseases, such as CAD, ACI and DM.

In most cases, the development of autoantibodies is caused by the overexpression of the particular corresponding antigens [20–22]. The expression level of adiponectin itself is negatively correlated with obesity, CAD and metabolic disorders [5,6]. Namely, the adiponectin level decreases during the progression of these diseases. On the other hand, the autoantibodies increased in patients with atherosclerosis-related diseases, such as ACI, AMI and DM (Figures 1–3). This implies that the development of adiponectin-Abs did not simply result from antigen overexpression but may play a causal and suppressive role in the progression of atherosclerosis.

The concentration of adiponectin in the sera of healthy individuals ranges from 5–30 μg/mL [23], whereas the concentration of adiponectin autoantibodies may be much lower. Adiponectin can form a variety of multimer complexes, among which high-molecular weight multimers, consisting of 12–18 monomers, cause anti-inflammatory, anti-atherogenic and anti-diabetic effects [24,25]. If only multimers consisting entirely of active monomers can cause such effects, the function might be disturbed by the binding of the antibody to a single monomer subunit. Thus, a low autoantibody level may be able to affect adiponectin function.

Because adiponectin has a causal role in the progression of metabolic syndrome, DM, CAD and atherosclerosis, several trials using adiponectin as a therapeutic agent have been undertaken [26–28]. It may be necessary to take into account the presence of anti-adiponectin-Abs, not only in the diagnosis but also in the therapy of atherosclerosis-related diseases.

## Abbreviations

ACI: acute cerebral infarction
AMI: acute myocardial infarction
AUC: area under the curve
CAD: coronary artery disease
DM: diabetes mellitus
HD: healthy donor
ROC: receiver operating curve.
